# TRIM5α and TRIMCyp form apparent hexamers and their multimeric state is not affected by exposure to restriction-sensitive viruses or by treatment with pharmacological inhibitors

**DOI:** 10.1186/1742-4690-6-100

**Published:** 2009-11-03

**Authors:** Marie-Édith Nepveu-Traversy, Julie Bérubé, Lionel Berthoux

**Affiliations:** 1 Laboratory of retrovirology, University of Québec, Trois-Rivières, QC, G9A 5H7, Canada

## Abstract

Proteins of the TRIM5 family, such as TRIM5α and the related TRIMCyp, are cytoplasmic factors that can inhibit incoming retroviruses. This type of restriction requires a direct interaction between TRIM5 proteins and capsid proteins that are part of mature, intact retroviral cores. In such cores, capsids are arranged as hexameric units. Multiple lines of evidence imply that TRIM5 proteins themselves interact with retroviral cores as multimers. Accordingly, stabilization by crosslinking agents has revealed that TRIM5α and TRIMCyp are present as trimers in mammalian cells. We report here that TRIM5 proteins seem to form dimers, trimers, hexamers and multimers of higher complexity in mammalian cells. The hexameric form in particular seems to be the most abundant multimer. Multimerization did not involve disulfide bridges and was not affected by infection with restriction-sensitive viruses or by treatment with the known TRIM5 inhibitors arsenic trioxide, MG132 and cyclosporine A. We conclude that TRIM5 multimerization results from more than one protein-protein interface and that it is seemingly not triggered by contact with retroviral cores.

## Findings

TRIM proteins form a family with dozens of members, most of them bearing a tripartite motif composed of a RING, B-box and Coiled-coil domains [1]. Restriction of retroviruses by members of the TRIM5 subfamily of TRIM proteins, which comprises the primate proteins TRIM5α and TRIMCyp [2-4], is initiated by physical recognition of the incoming retrovirus by TRIM5 proteins. This interaction occurs within the first hours following virus entry [5] and involves determinants present in the N-terminal domain of the capsid proteins which constitute the retroviral outer core structure [6-8]. Retroviral capsid cores are assembled from hundreds of capsid proteins and the basic capsomer is a hexamer [9-11]. Restriction necessitates capsid proteins of the incoming retrovirus to be correctly maturated by the retroviral protease [12,13]. This is a required step for the core to adopt its final structure. In addition, mutations that affect the stability of the retroviral core interfere with the efficiency of restriction [12,13]. Virus-free capsid proteins, which do not multimerize to form cores, do not interact with TRIM5 proteins in cells [14]. That TRIM5-mediated restriction requires assembled retroviral cores brings the question of whether TRIM5 proteins themselves must be present as multimers. TRIM proteins are known to homomultimerize through their coiled-coil domain [1], which is required for restriction [15]. TRIM5 proteins from different species can interact with each other and in doing so can interfere with each other's restriction activity [16]. TRIM5α has also been shown to trap incoming retroviral particles inside cytoplasmic bodies, which further suggests that TRIM5 proteins interact with their targets as multimers [17]. TRIM5α and TRIMCyp have been stabilized as trimers by treatment with cross-linking agents [18-23]. Some undefined higher-order multimers have been occasionally observed [18,19]. The relevance of trimerization was confirmed by the fact that modified TRIMCyp, in which the coiled-coil domain is substituted by that of a trimeric heterologous protein, restricted HIV-1, although at much lower levels than wild-type TRIMCyp did [19]. A recombinant TRIM5 protein expressed in insect cells was observed as dimers [21] and minor amounts of dimeric TRIM5α have been observed in cells [23]. However, dimerization/trimerization of TRIM5 proteins fails to explain the formation of cytoplasmic bodies or the sequestration of incoming restricted virus in such structures. Thus, we analyzed TRIM5α/TRIMCyp multimerization in the presence or absence of restriction-sensitive viruses and upon treatment with various drugs that inhibit the restriction process.

**Figure 1:**
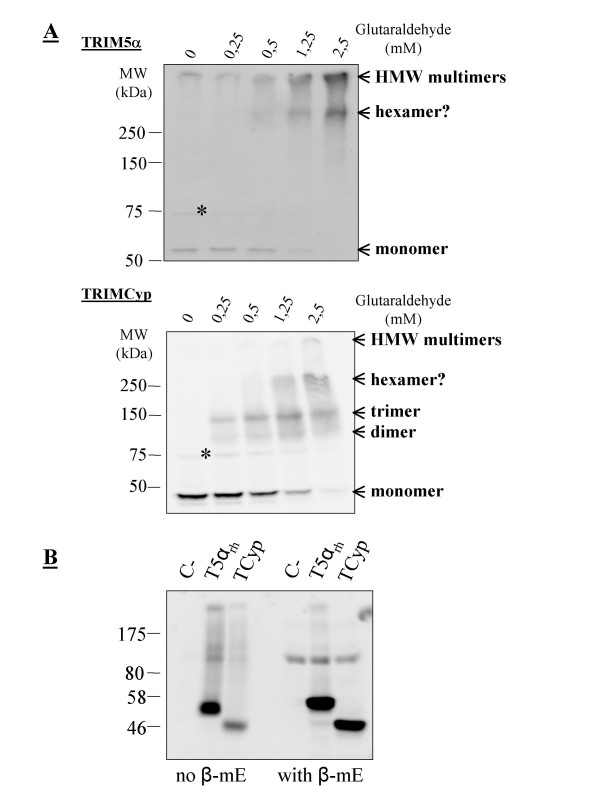
**Multimerization profiles of TRIM5α and TRIMCyp**. **A**, 0.5% NP40 lysates were prepared from *Mus dunni *tail fibroblast cells (MDTFs) stably expressing FLAG-tagged TRIM5α_rh _or owl monkey TRIMCyp. The soluble fraction of each lysate was divided in aliquots that were treated for 5 min with the indicated glutaraldehyde concentrations before proteins were denatured by boiling in the presence of SDS. Proteins were then separated on an 8% polyacrylamide gel, transferred to a nitrocellulose membrane, and probed with a rabbit anti-FLAG antibody (Cell Signaling). The apparent multimeric states are indicated on the right as deduced from the size of the bands. The star indicates an unspecific protein cross-detected by the FLAG antibody. **B**, Lysates were prepared from HeLa cells stably transduced with the same constructs as above and in the absence or presence of 100 μM β-mercaptoethanol as indicated.

We first analyzed TRIM5 multimerization in stably transduced *Mus dunni *tail fibroblast (MDTF) cell lines [24]. Multimers were stabilized by treatment with glutaraldehyde as first described by Mische and collaborators [23]. Surprisingly, TRIM5α_rh _was not present as a trimer in these cells. Rather, we observed a band with a size in the 300-400 kDa range (Fig. 1), and subsequent experiments that used a different molecular weight marker confirmed this apparent weight. Since the TRIM5α_rh _monomer migrates at 55 to 60 kDa, this multimer may be a hexamer. Higher-order multimers were also seen but their size could not be estimated. These high molecular weight multimers were present in the stacking gel when they were seen; and in some experiments they were found to have barely penetrated the acrylamide. We cannot exclude that they might be aggregates rather than genuine higher-order assemblies of TRIM5α. TRIMCyp was found in MDTF cells as dimers and trimers and also as higher-order multimers that included a band slightly heavier than the 250 kDa marker (Fig. 1). Since monomeric TRIMCyp migrates at about 45 kDa, the multimer seen is most likely a hexamer (although the migration pattern of multimeric complexes might be different from those of linear proteins). Higher amounts of glutaraldehyde were required to reveal the presence of hexamers and higher-order multimers, compared with dimers or trimers. Thus, TRIM5α and TRIMCyp can have distinct multimerization profiles despite both being fully active in MDTF cells. They also share the capacity to form apparent hexamers. Because coiled-coil domains can dimerize through the formation of covalent disulfide bridges between cysteine residues in some instances [25], we performed a Western blot analysis of TRIM5α and TRIMCyp in reducing and nonreducing conditions. In the absence of β-mercaptoethanol, both TRIM5α and TRIMCyp were less easily detected, but migrated at the expected size; and no dimer or more complex multimers were visible (Fig. 1B), with the exception of very high molecular weight structures which seemed to be present in higher amounts compared to the reducing conditions. Thus, it appears that disulfide bridges do not induce TRIM5 protein dimers, trimers or hexamers, but perhaps they are involved in the formation of non-specific aggregates.

**Figure 2:**
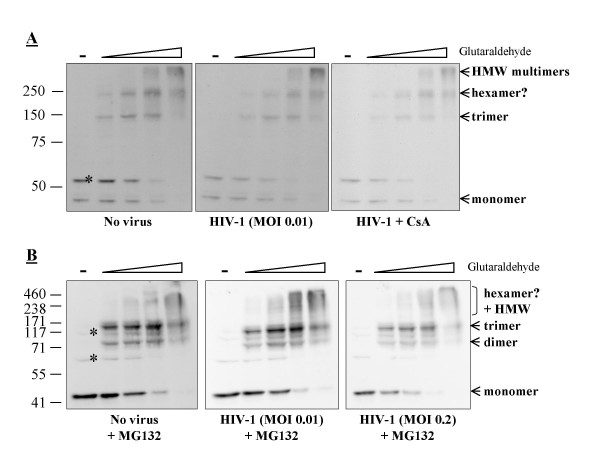
**Multimerization of TRIMCyp in cells infected by HIV-1**. **A**, MDTF-TRIMCyp cells were challenged with an HIV-1 vector expressing GFP, at a dose leading to infection of about 1% of the cells and either in the presence or not in the presence of 5 μM cyclosporine A (Sigma). After 6 hours of infection, cells were lysed in presence of increasing glutaraldehyde concentrations as in Fig. 1. Western blot analysis of FLAG-tagged proteins was performed as above. **B**, the experiment was repeated in the presence of 1 μM MG132 (Sigma) and using two different virus doses. The stars indicate cellular proteins cross-detected by the FLAG antibody as evidenced by analysis of lysates from parental cells (not shown).

To investigate the possibility that TRIMCyp multimerization was induced or modulated by exposure to a restriction-sensitive virus, we repeated the glutaraldehyde crosslinking assay after 6 hours of continuous infection with TRIP-CMV-GFP, which is an HIV-1 vector encoding GFP [24,26]. Approximately 1% of the MDTF-TRIMCyp cells were infected in these conditions, versus more than 50% of the same cells not expressing TRIMCyp (not shown). Thus, TRIMCyp restriction activity was not saturated at this multiplicity of infection (MOI), yet cells were exposed to large amounts of HIV-1 virions in order to maximize the frequency of TRIMCyp:capsid interaction. However, HIV-1 infection did not noticeably modify the relative amounts of TRIMCyp trimers, hexamers and higher-order multimers (Fig. 2A). We repeated the experiment in the presence of cyclosporine A (CsA), which completely abrogates the restriction mediated by TRIMCyp as it binds to the same CypA domain that recognizes HIV-1 capsid proteins [27,28]. CsA treatment, however, had no effect on TRIMCyp multimerization profiles, further implying that multimerization was independent of specific virus recognition.

It was recently reported that TRIM5α and TRIMCyp are degraded in a proteasome-dependent pathway following infection with a restriction-sensitive retrovirus [29]. Thus, it was conceivable that in our previous experiment HIV-1 modulated the multimerization of only a part of the cellular TRIMCyp proteins which were then degraded by the proteasome. To address that possibility, we repeated the experiment in the presence of the proteasomal inhibitor MG132, thereby preventing virus-induced TRIMCyp targeting to the proteasome (not shown). In addition we infected with a higher dose of the HIV-1 vector, leading to 20% infected cells. TRIMCyp restriction activity was significantly saturated at this MOI, implying that most TRIMCyp proteins that were restriction-competent at the time of infection were indeed engaging incoming HIV-1 [24]. However, MG132 did not appreciably modify the multimerization profile of TRIMCyp in the absence or presence of HIV-1 (Fig. 2B). Like before, dimers, trimers and higher-order multimers were formed. The band corresponding to putative hexamers was less well-defined compared with previous experiments, but this is probably due to technical reasons unrelated to the effects of MG132 on TRIM5.

**Figure 3:**
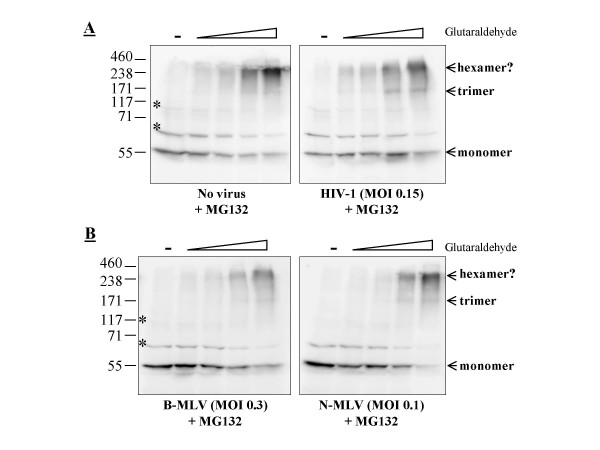
**Multimerization of TRIM5α_AGM _is not modulated by infection with restriction-sensitive viruses**. **A**, MDTF cells expressing TRIM5α_AGM _were either infected or not infected with HIV-1 for 6 hours, using a virus dose leading to about 15% infected cells and in the presence of 1 μM MG132. Crosslinking assays were done as before. **B**, MDTF-TRIM5α_AGM _cells were infected with identical amounts (as normalized by titration on parental cells) of B-MLV or N-MLV-derived vectors expressing GFP in the presence of MG132. 30% and 10% of the cells were infected (GFP-positive), respectively, by B-MLV-GFP and N-MLV-GFP, as seen by flow cytometry 2 days later.

We used MDTF cells expressing TRIM5α cloned from Vero cells (African green monkey) [24] to investigate whether, unlike that of TRIMCyp, TRIM5α multimerization could be modulated upon infection with a restricted virus. This orthologue of TRIM5α decreases HIV-1 replication by about 100-fold [30] and also inhibits the N-tropic strains of the murine leukemia virus (MLV), although to a smaller extent (10-fold or less) [24]. As in Fig. 2, we challenged these cells with restricted (HIV-1 and N-MLV) or non-restricted (B-MLV) viruses at relatively high doses and in presence of MG132 (Fig. 3). Under these conditions, the inhibition of N-MLV by TRIM5α_AGM _was lower than previously observed, a likely consequence of the MG132 treatment and of the high MOI (not shown). TRIM5α_AGM _formed apparent trimers and hexamers in these cells but no dimers were observed (Fig. 3A), nor did we see multimers of very high molecular weight in this particular experiment. Challenges with the different viruses had little effect on the multimerization pattern. The relative number of hexamers stabilized at the highest glutaraldehyde concentration used, decreased slightly in cells infected with one of the restricted viruses (HIV-1; Fig. 3A), but increased slightly in cells infected with the other restricted virus (N-MLV; Fig. 3B). No notable differences were found at the other glutaraldehyde concentrations. Data from Fig. 2 and 3 together suggest that the multimerization of TRIM5 proteins is not modulated by retroviral infections. A caveat in these experiments, however, is that the percentage of TRIM5 proteins actually engaged in the restriction process at any given time is not known. Even at high multiplicities of infection, it is still possible that modulation of multimerization occurs at levels undetectable in our assays.

**Figure 4:**
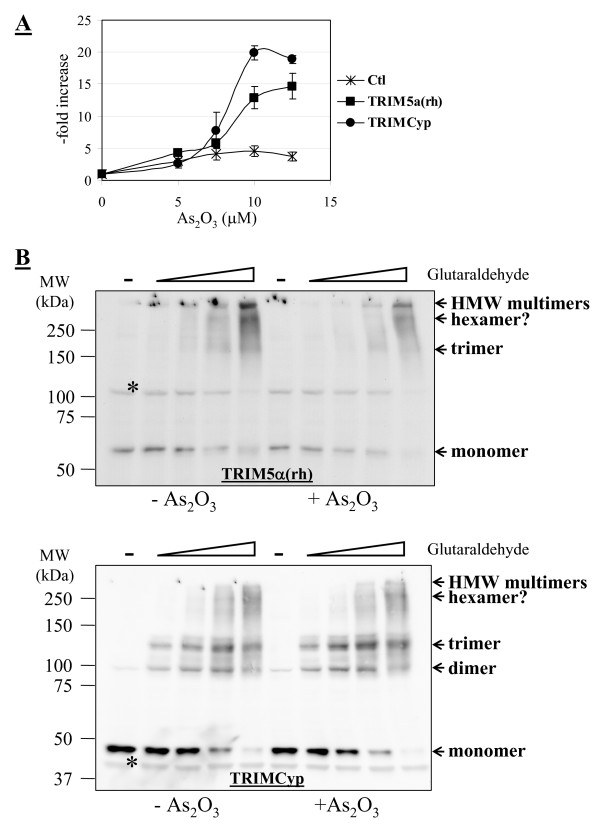
**As_2_O_3 _does not modify the multimerization of TRIM5α and TRIMCyp**. **A**, human HeLa cells stably expressing TRIM5α_rh _and TRIMCyp, or control untransduced cells, were challenged with an HIV-1 vector expressing GFP at virus doses leading to about 1% infected (GFP-positive) cells in the absence of drug. Infections were performed in the presence of increasing As_2_O_3 _concentration (x-axis) and for 10 hours, after which supernatants were replaced with fresh medium to avoid As_2_O_3_-related toxic effects. 2 days after infection, the % of cells expressing GFP were determined by flow cytometry analysis. Results were expressed as -fold increase compared with the untreated control. The averages from triplicate infections with standard deviations are shown. **B**, glutaraldehyde assays were performed exactly as before and in the absence or presence of 10 μM As_2_O_3_.

Arsenic trioxide (As_2_O_3_) inhibits the restriction activity of TRIM5 proteins in a virus-independent, TRIM5 orthologue-independent, cell type-dependent manner [30,31]. The mechanism of action of this drug on TRIM5 proteins is at present unknown. Thus, it was of interest to analyze whether it could affect the capacity of TRIM5 proteins to multimerize. We found that As_2_O_3 _did not affect TRIM5-mediated restriction in MDTF cells (not shown), and thus we used human HeLa cells for this particular experiment. As expected, stable expression of TRIM5α_rh _and of TRIMCyp in HeLa cells resulted in an approximately 100-fold reduction in permissiveness to transduction with an HIV-1 virus expressing GFP (not shown). We found that both restriction activities were partly suppressed by As_2_O_3 _treatment (Fig. 4A). More precisely, a short (10 hours) treatment with As_2_O_3 _at the time of infection increased permissiveness to HIV-1 by up to 15-fold in cells expressing TRIM5α_rh _and 20-fold in cells expressing TRIMCyp, while having a smaller, "background" effect of about 4-fold in the control untransduced cells. Crosslinking assays yielded slightly different results in HeLa cells compared with what had been observed in MDTF cells. TRIM5α_rh _did not dimerize but trimers were visible, as well as apparent hexamers and higher-order multimers (Fig. 4B, upper panel). TRIMCyp was found as dimers, trimers, and hexamers (Fig. 4B, lower panel). An additional band migrating faster than the hexamer was visible and could be a pentamer. In both cases, the experiment was done in the absence or presence of As_2_O_3_; and no differences were observed. Therefore, arsenic trioxide does not influence the multimeric state of TRIM5α and TRIMCyp.

We find that in addition to the dimeric and trimeric forms previously described, TRIM5α and TRIMCyp can form apparent hexamers and more complex multimers. Why discrete hexamers were not previously seen by others is probably only related to the difficulty of resolving high molecular weight complexes in acrylamide gels, although we cannot totally exclude that the C-terminus FLAG tag used in our constructs may somehow interfere with protein multimerization. Because of low expression levels in mammalian cells, it is not possible at this point to perform the biochemical experiments that would be needed to ascertain that the various multimers seen here are composed of TRIM5 proteins only. For instance, a trimer of TRIM5 could associate with a heterologous cellular protein, yielding a band resembling a TRIM5 hexamer. Thus, other approaches will be needed. The hexamer model is obviously appealing because capsid proteins are themselves organized as hexamers in mature retroviral cores. Thus, a hexamer of TRIM5 proteins could be needed to recognize a retroviral capsomer. Formation of dimers, trimers and hexamers, however, does not seem to be triggered by contact with a restricted retrovirus. It remains possible that the nature and number of some specific higher order multimers not resolved in our gels could be modulated during the restriction process. Not surprisingly, the coiled-coil domain of TRIM5 proteins has been found to be required for the formation of trimers [18,23]. However, this does not imply that a single protein:protein interface present in this domain is responsible for the various multimeric forms observed. Rather, it is more likely that one interface would lead to dimerization and another one to trimerization; together they would be responsible for hexamerization. Perhaps yet other determinants within TRIM5α and TRIMCyp lead to the formation of very high molecular weight multimers. Consistent with the existence of more than one molecular site of TRIM5:TRIM5 interactions, Li and Sodroski have recently reported that point mutants in the B-Box domain show normal multimerization patterns in crosslinking assays while being less efficient at engaging in protein:protein interactions through co-immunoprecipitation assays [22]. Regardless of what the exact molecular mechanism of TRIM5 multimerization is, our data suggest that TRIM5 multimerization is complex but that formation of low molecular weight multimers is not influenced by contact with a restricted retrovirus.

## Competing interests

The authors declare that they have no competing interests.

## Authors' contributions

MÉNT, JB and LB designed the study. MÉNT and JB performed the experiments. LB drafted the manuscript. All authors read and approved the final draft.
